# Multi‐omic approaches to defining disease heterogeneity, subtypes, and progression

**DOI:** 10.1002/alz.086461

**Published:** 2025-01-03

**Authors:** David A. Bennett

**Affiliations:** ^1^ Rush University, Chicago, IL USA

## Abstract

**Background:**

The aging and dementia field has long been interested in understanding disease heterogeneity, subtypes, and progression. Work has progressed from clinical, to neuroimaging to biomedical devices to neuropathological data, and now brain and blood omic data.

**Method:**

The AMP‐AD consortium generated and/or annotated genomic, epigenomic, transcriptomic, proteomic, and metabolomic data from brain and/or blood from thousands of study participants and patients across the 8 teams. They include women, men, non‐Latino white and black, and Latinos, covering the full spectrum of clinical and neuropathologic traits and diseases. All data were deposited in the AD Knowlege Portal (www.synapse.org) and made available to the wider aging and dementia research community in an open science environment.

**Result:**

The omic data has been leveraged by members of the AMP‐AD teams, the SAGE bionetworks team which manages the Portal, and the wider research community to address disease heterogeneity, subtypes, and progression at the molecular level. New computation tools and approaches were developed and/or adapted from the cancer space as such deep and varied brain omic data were not previously available from humans. Initial studies used a single layer of data. Later studies integrated two or more layers of data. Ongoing work is now bridging the gap to project blood omic data to molecular trajectories in the same humans. Examples of the work across this spectrum will be presented.

**Conclusion:**

The data generated and/or annotated in first decade of AMP‐AD is now defining disease heterogeneity, subtypes, and progression at the molecular level in the brain and beginning to bridge the gap to data obtainable from living humans. It is hoped that this will move the field closer to a time when precision medicine for brain diseases can target brain molecular pathways.

